# Drug Transport in a Liquid-Crystalline Supramolecular Hydrogel: Diffusion Mechanisms Revealed by PGSE NMR

**DOI:** 10.3390/pharmaceutics18050592

**Published:** 2026-05-12

**Authors:** Wei Wang

**Affiliations:** 1 Department of Chemistry, University of Bergen, 5020 Bergen, Norway; wei.wang@uib.no; Tel.: +47-5558-3355; 2 Centre for Pharmacy, University of Bergen, 5020 Bergen, Norway

**Keywords:** diffusion, liquid crystalline, hydrogel, PGSE NMR, C18ADPA

## Abstract

**Background/Objectives:** Supramolecular hydrogels formed by low-molecular-weight gelators present a chemically heterogeneous transport environment whose molecular-scale dynamics remain poorly understood. This study aimed to investigate how drug physicochemistry governs transport within a liquid-crystalline C18ADPA hydrogel at the molecular scale. **Methods:** Pulsed-field gradient NMR spectroscopy was used to measure self-diffusion coefficients of five model drugs (5-fluorouracil, acetylcholine, paracetamol, prednisolone, and amphotericin B) spanning a broad range of size, polarity, and charge state, in both free solution and the hydrogel matrix at pH 5.37. **Results:** Observed drug diffusion coefficients deviated substantially from classical obstruction theory predictions, demonstrating that transport is governed by host–guest chemical affinity rather than molecular size. The three water-soluble drugs exhibited bimodal diffusion, with relative amplitudes providing a direct estimate of bound and free drug fractions. Prednisolone co-diffused with the gelator scaffold, consistent with hydrophobic bilayer partitioning, while amphotericin B diffused at rates consistent with the structured interfacial water layer. The gel pH (5.37) emerged as an active determinant of transport: drug charge states at this pH from permanent cation (acetylcholine) to near-zwitterion (amphotericin B) correlated directly with the observed transport behavior. The near-zwitterionic character of amphotericin B at pH 5.37, arising from its carboxyl pKa (~5.5), suggests a previously unreported electrostatic interfacial trapping mechanism. **Conclusions:** The liquid-crystalline bilayer architecture creates chemically distinct microdomains that selectively recruit drugs based on hydrophobicity, hydrogen-bonding capacity, and pH-dependent charge state, providing a molecular-scale framework for rational formulation design in supramolecular drug delivery.

## 1. Introduction

The architectural mimicry of the extracellular matrix has long been a central objective in soft-matter engineering. Supramolecular hydrogels, also known as low-molecular-weight hydrogels (LMWGs), have emerged as a distinct class of materials that fundamentally diverge from polymer networks [1]. These gels form through the hierarchical self-assembly of small molecules into high-aspect-ratio fibers, tubes, or ribbons that entrap more than 90 wt% water through capillary forces and surface tension [2]. Unlike polymer hydrogels, whose structures are fixed by permanent cross-links, supramolecular networks are stabilized by reversible non-covalent interactions such as hydrogen bonding, π–π stacking, and hydrophobic effects. This dynamic bonding imparts “living” material characteristics, stimuli responsiveness, shear-thinning injectability, and autonomous self-healing, that operate on biologically relevant timescales [3]. As a result, they have become widely explored for regenerative medicine, 3D bioprinting, and precision drug delivery [4]. A single physical parameter governs the drug performance of these hydrated scaffolds: mass transport [5]. In drug delivery, the objective is often to retain and modulate the release of therapeutic cargo. This duality underscores the core design paradox: the network must be sufficiently permeable yet restrictive enough to control release kinetics [6].

Despite the central importance of diffusion, quantifying solute transport in soft, dynamic supramolecular networks remains challenging. Classical continuum models, such as the Ogston [7], Mackie–Meares [8], or free-volume theories [9], treat hydrogels as static arrangements of inert steric obstacles. These models, developed largely for covalent polymer meshes, attribute mobility reduction solely to pore tortuosity and solvent drag [10]. However, they fail to capture transport in supramolecular systems, where the matrix behaves not as a passive sieve but as a chemically active, fluctuating energy landscape [11,12]. The high interfacial area of the self-assembled fibers creates domains in which solute mobility is dominated by electrostatic anchoring, hydrophobic partitioning, and hydrogen-bond interactions rather than simple size exclusion. Moreover, supramolecular fibers undergo continuous breaking, reptation, and reformation on timescales comparable to solute motion, giving rise to anomalous diffusion behaviors that macroscopic release experiments, such as elution studies or Franz diffusion cells, cannot resolve [13]. These bulk measurements effectively treat the hydrogel as a “black box,” yielding only an apparent diffusion coefficient that conflates pore connectivity, viscosity, and binding events.

To engineer next-generation programmable delivery vehicles, it is therefore necessary to move beyond curve-fitting macroscopic release profiles and instead interrogate solute dynamics at the microscopic scale. PGSE NMR, commonly implemented as Diffusion-Ordered Spectroscopy (DOSY), provides this capability. PGSE NMR measures self-diffusion over defined diffusion times (Δ), enabling the deconvolution of molecular populations based on hydrodynamic mobility. Importantly, it allows simultaneous observation of the solute, the solvent, and the gelator fibers, offering a holistic picture of multicomponent dynamics [14].

**Figure 1:**
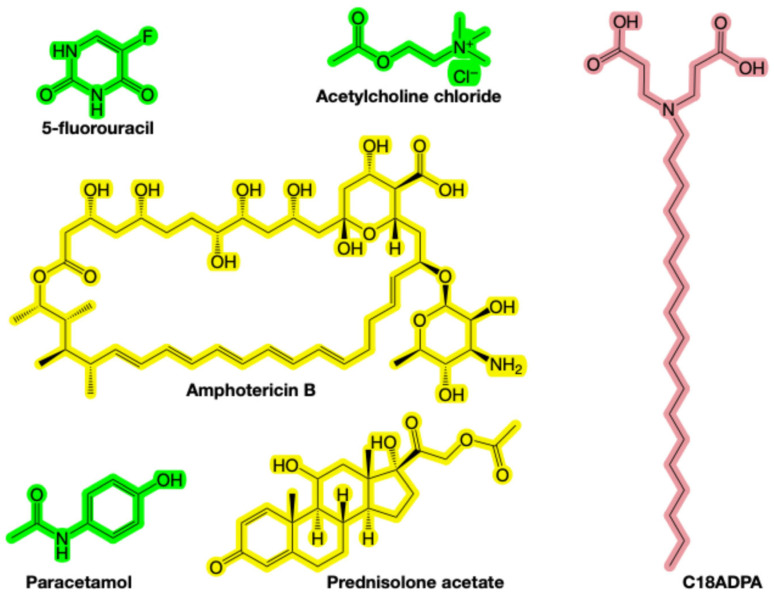
The chemical structures of C18ADPA (red), 5-fluorouracil, acetylcholine chloride, amphotericin B, paracetamol, and prednisolone acetate. Three molecules highlighted in green are considered highly water-soluble, whereas the two molecules highlighted in yellow are classified as poorly water-soluble.

Here, we investigate how supramolecular hydrogel architecture and interfacial chemistry regulate drug diffusion. We employ C18ADPA, a multi-headed amphiphilic surfactant synthesized previously in our laboratory (structure in Figure 1) [15,16,17,18]. C18ADPA self-assembles into entangled fibrillar networks within a narrow, physiologically relevant pH range, presenting hydrophobic alkyl chains and hydrophilic headgroups at the water–fiber interface and thereby generating a chemically heterogeneous landscape [17,19]. To probe how guest–host chemistry influences transport, we selected five model drugs (5-fluorouracil, acetylcholine, paracetamol, prednisolone, and amphotericin B) that span a broad range of size, polarity, rigidity, and amphiphilicity. Critically, these drugs also differ in their ionization states at the gel pH of 5.37: acetylcholine is permanently cationic (quaternary ammonium); 5-fluorouracil (pK_a1_~7.5) and paracetamol (pK_a_~9.5) are neutral; prednisolone has no ionizable groups; and amphotericin B (pK_a1_~5.5, pK_a2_~10) is zwitterionic near the gel pH. This chemical diversity enables a systematic deconvolution of electrostatic, hydrophobic, and hydrogen-bonding contributions to transport. Using high-resolution PGSE NMR, we measured drug diffusion in free solution and within the C18ADPA hydrogel. By varying solute chemistry while maintaining a constant host architecture, we deconvolute the respective contributions of steric confinement, hydrodynamic coupling, and thermodynamic partitioning to the overall diffusion behavior.

## 2. Materials and Methods

### 2.1. Materials

Acetylcholine, 5-fluorouracil, amphotericin B, paracetamol, and prednisolone acetate were provided as gifts from the laboratory of Prof. Yuji Wang (Capital Medical University, China), and the chemical structures of these drugs are presented in Figure 1. D_2_O was 99.9 atom% D (Merck, Darmstadt, Germany), NaOH and HCl were of analytical grade and used as received. The drug samples received as gifts were used without further purification. Their purity was confirmed by ^1^H NMR prior to diffusion experiments, based on clean single-component spectra with no spurious peaks in the regions used for diffusion coefficient determination. Milli-Q water was used throughout all experiments. Zwitterionic amphiphile 3,3′-(octadecylazanediyl) dipropionic acid (C18ADPA) was synthesized based on our previous publications [17,19].

### 2.2. Preparation of Drug-Loaded Hydrogels

The aqueous solution of C18ADPA forms a hydrogel when its concentration reaches approximately 2 wt%. The preparation method for blank C18ADPA samples is detailed in our previous publications.

To load drugs for NMR measurements, the blank gel undergoes a freeze-drying process to yield a xerogel. Subsequently, the xerogel (0.02 g) and D_2_O (1.00 g) are mixed with varying quantities of drugs in a vial. The drug loading in the hydrogels is as follows, 5-fluorouracil: 0.0106 g; acetylcholine chloride: 0.0033 g; amphotericin B: 0.0020 g; paracetamol: 0.0103 g; prednisolone acetate: 0.0027 g.

**Figure 2:**
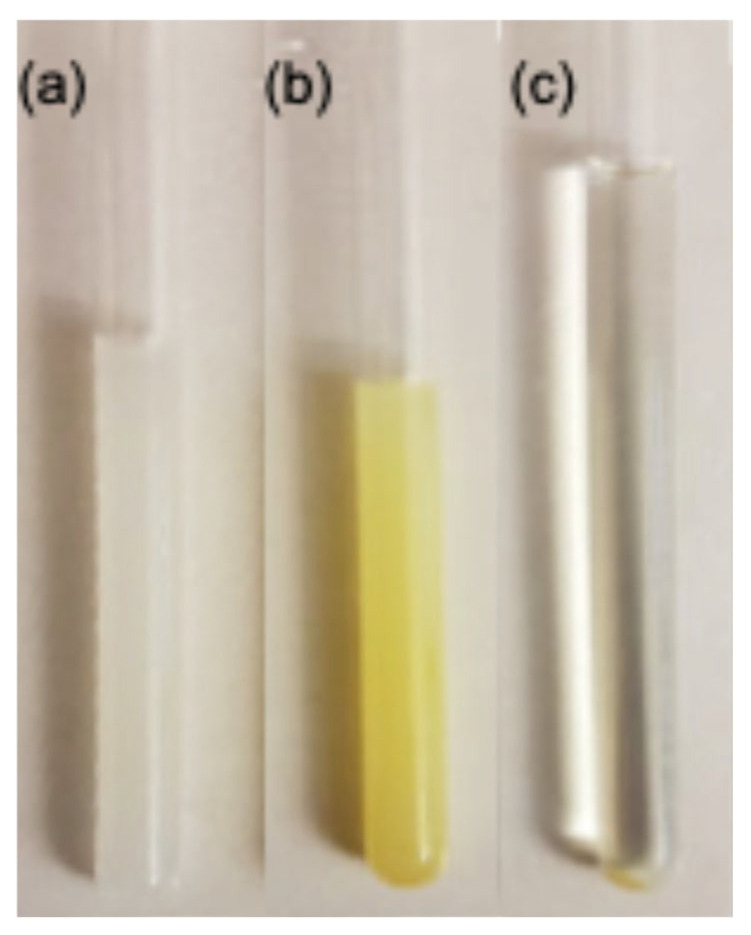
The sample images: (**a**) pure gel; (**b**) amphotericin B-loaded gel; (**c**) amphotericin B solution.

The pH of all samples was adjusted to 5.37 ± 0.05 using dilute HCl or NaOH prior to heating. This value corresponds to the well-characterized gelation window of C18ADPA established in our previous work, at which both the carboxylic acid groups and the tertiary amine of C18ADPA are ionized, yielding the zwitterionic form required for stable lamellar self-assembly [17,19]. The mixture is stirred and heated to 60 °C in a water bath to form a viscous solution. This solution is then transferred into a WILMAD-528PP-5mm NMR tube and placed back in the water bath. After a 5-min heating period, the water bath is allowed to cool to room temperature. Parallel to this process, drug solutions (without C18ADPA) were prepared at the same pH using an identical procedure. Subsequently, the diffusion of drugs is measured for both the hydrogel samples and the drug solutions. Figure 2 illustrates the visual characteristics of pure gel, drug-loaded gel, and drug solution, utilizing amphotericin B as a representative example. The drug-loaded gel is homogenously yellow (Figure 2b), indicating enhanced solubility of amphotericin B in C18ADPA gel. Conversely, the drug solution exhibited near-transparency, with a precipitate visible at the bottom of the NMR tube (Figure 2c) due to the limited solubility of amphotericin B in aqueous solutions. The variation in drug concentrations across different samples was determined based on the following considerations. First, the amount added was guided by the intrinsic solubility of each compound in water. Second, for drugs with relatively low aqueous solubility, we further evaluated the impact of the added quantity on the NMR signal to ensure that the drug reached a saturated concentration. The same amount of drug was introduced into both aqueous solutions and gel samples. The gel matrix exhibits a solubilizing effect; therefore, in aqueous solutions, a small amount of precipitation may occur. Such precipitates, however, do not interfere with NMR measurements. To assess reproducibility, selected samples (5-fluorouracil- and prednisolone-loaded gels) were prepared in duplicate from independent gelation batches. The reported diffusion coefficients for these duplicates agreed within 5%, confirming the robustness of the preparation protocol. The consistency of the internal water reference across all samples (H_2_O ≈ 1.87–1.89 × 10^−9^ m^2^/s in solution; see Table 1) confirms stable sample conditions and reproducibility of the experimental setup across batches.

**Table 1:** Diffusion coefficients (m^2^/s) obtained from the fitting in Figure 3.

Component	5-Fluorouracil	Acetylcholine	Paracetamol	Prednisolone	Amphotericin B
**D_Drug_**	7.714 × 10^−10^	7.025 × 10^−10^	5.811 × 10^−10^	4.297 × 10^−10^	2.950 × 10^−10^
**D_H2O_**	1.873 × 10^−9^	1.893 × 10^−9^	1.848 × 10^−9^	1.894 × 10^−9^	1.887 × 10^−9^
**R_H_ (nm)**	0.23	0.25	0.31	0.41	0.60

**Figure 3:**
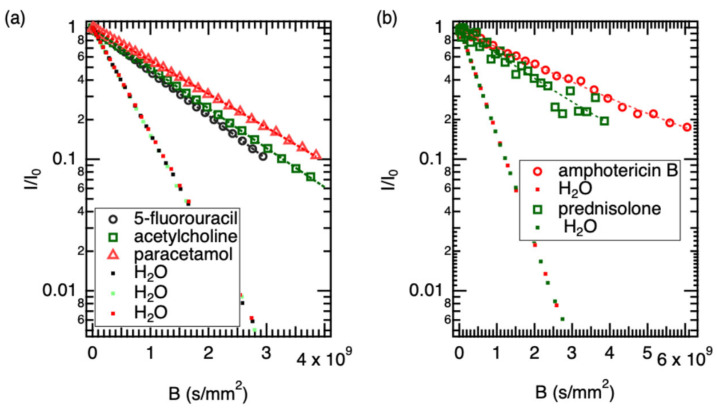
The data and monomodal fitting for (**a**) 5-fluorouracil, acetylcholine, and paracetamol, (**b**) for amphotericin B and prednisolone. The dots are the diffusion curve of water in both panels.

### 2.3. The Diffusion Measurements

The diffusion of the drug molecules was determined by PGSE NMR. PGSE NMR was carried out on a Bruker Avance III 500 WB spectrometer operating at 500 MHz for protons. The spectrometer was equipped with a commercial NMR probe (diff30) capable of producing magnetic field gradient pulses up to 17 T/m in the z-direction. The measurements were performed at 298 K (25 °C; this temperature was chosen to ensure gel stability and consistency with previously reported structural and diffusion data for this system, as the C18ADPA hydrogel undergoes a lamellar-to-wormlike micelle transition above 43 °C) using a stimulated echo sequence with bipolar field gradient pulses (diffSteBp). The pulse sequence of diffSteBp consists of a three-pulse sequence: 90–T_1_-90–T_2_-90–T_3_-acquisition, where T_2_ represents the mixing time during which longitudinal magnetization storage occurs. The diffusion encoding is achieved through bipolar gradient pulse pairs applied during T_1_ and T_3_ periods. Each gradient encoding block consists of two gradient pulses of equal magnitude but opposite polarity (±g), separated by a 180° refocusing pulse. The first bipolar gradient pair (duration δ each, set to 1 ms) is applied after the first 90° pulse, followed by the second bipolar pair after the third 90° pulse, with a diffusion time Δ (set to 20 ms) between the centers of the gradient pairs. The results of diffusional NMR are presented in the Supporting Information Figures S1–S5 for all five drugs.

The signal attenuation was analyzed by the Stejskal-Tanner equation [20]:

(1) $$S\left(t\right)=S\left(0\right)e^{-\gamma^{2}g^{2}\delta^{2}D\left(\Delta -\delta /3\right)}$$

where γ is the magnetogyric ratio, g is the gradient strength, δ is the gradient pulse duration, D is the diffusion coefficient, and Δ is the diffusion time. For systems with multiple diffusion components, the equation was extended to include contributions from each component with their respective diffusion coefficients. Data processing was conducted using Bruker TopSpin 4.0.6 software and spectral plots were generated using MestReNova v14, employing auto-processing of FID curves followed by manual peak integration at 95% confidence intervals to determine signal attenuation and calculate diffusion coefficients using the model I = Σ_j_ I_j_e^(−BD_j_). Here, B is γ^2^g^2^δ^2^(Δ − δ/3), D_j_ is the diffusion coefficient corresponding to the mode j, and I_j_ is the intensity of the corresponding mode.

## 3. Results

### 3.1. The Diffusion of the Drugs and Water in the Solution Samples

Figure 3 shows the fitting of diffusion data for five drugs: (a) three well-dissolved drugs and (b) two sparingly soluble drugs. All diffusion profiles were successfully fitted using a monomodal diffusion model, indicating that a single dominant diffusion mechanism governs the transport process under the experimental conditions. However, the quality of the fit varied among the drugs. For the water-soluble compounds, the experimental data aligned closely with the model predictions.

The internal water reference consistently showed diffusion values close to 1.89 × 10^−9^ m^2^/s, confirming stability in sample viscosity. The solute diffusion coefficients followed the expected inverse trend with molecular weight (MW), ranging from 7.71 × 10^−10^ m^2^/s for the lightest species (5-Fluorouracil) to 2.95 × 10^−10^ m^2^/s for the heaviest (Amphotericin B).

A distinct hydrodynamic variation was observed between acetylcholine (146 Da) and paracetamol (151 Da). While their molecular weights differ by less than 4%, their diffusion coefficients diverge significantly (7.03 × 10^−10^ m^2^/s vs. 5.81 × 10^−10^ m^2^/s, respectively). This behavior can be rationalized by the nature of their solvation shells. Acetylcholine, despite being a cation, possesses a quaternary ammonium group surrounded by three methyl moieties. This creates a “soft” cationic center characterized by hydrophobic hydration, where water molecules are less tightly bound compared to strong hydrogen-bonding sites. This allows for relatively rapid translational mobility. In contrast, paracetamol is a rigid aromatic system functionalized with both a phenolic hydroxyl and an amide group. These moieties act as potent hydrogen bond donors and acceptors, creating a “sticky” interaction with the aqueous solvent. This strong solvent drag increases the effective hydrodynamic friction, causing paracetamol to diffuse significantly slower than the similarly sized acetylcholine.

Prednisolone displayed a diffusion coefficient of 4.297 × 10^−10^ m^2^/s. This reduction aligns with the rigid, tetracyclic steroidal core, which presents a larger cross-sectional area to the solvent compared to the single-ring aromatics. Amphotericin B exhibited the lowest diffusivity (2.950 × 10^−10^ m^2^/s). Given its high molecular weight (924 Da) and amphiphilic polyene structure, this value is consistent with the molecule adopting a large hydrodynamic volume. While amphotericin B is prone to aggregation, the observed D value suggests that under these specific experimental conditions, it remains sufficiently soluble to be detected, likely existing as a monomer or small oligomer equilibrium.

The hydrodynamic radii (r_H_) were subsequently derived using the Stokes–Einstein equation:

(2) $$r_{H}=\frac{k_{B}T}{6\pi \eta D}$$

where the symbols follow standard physical notation, and the viscosity of the solvent is taken as 1.232 mPa s for D_2_O. The results are presented in Table 1.

A general inverse correlation was observed between molecular weight and diffusivity, consistent with the discussion above. The notable exception—acetylcholine diffusing faster than the similarly sized paracetamol—reflects the contrasting solvation character of the two molecules, as described in the preceding section.

The r_H_ calculated for prednisolone (0.41 nm) is consistent with its rigid, tetracyclic steroidal backbone. The molecule tumbles as a relatively oblate object, creating higher friction than the smaller spherical aromatics. For amphotericin B, the determined radius of 0.60 nm provides insight into its solution state. Amphotericin B is an amphiphilic polyene known to self-assemble into dimers or oligomers in aqueous environments. A monomeric radius for a molecule of this mass (924 Da) would be expected to be slightly smaller (approx. 0.50–0.55 nm). The observed value of 0.60 nm suggests that under the specific concentration and conditions of this study, amphotericin B likely exists in a rapid equilibrium between monomeric and small oligomeric states, preventing the observation of discrete aggregate peaks but resulting in a time-averaged D value lower than that of a pure monomer.

### 3.2. The Diffusion of the Drugs, C18ADPA, and Water in the Gel Samples

**Figure 4:**
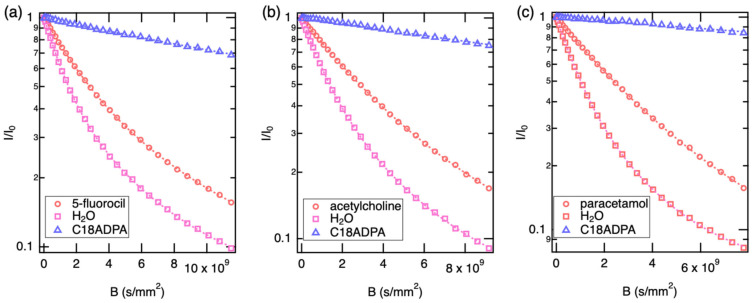
The Stejskal–Tanner plots of (**a**) 5-fluorouracil, (**b**) acetylcholine and (**c**) paracetamol diffusion in gel samples. The diffusion data of the drugs and water were fitted with bimodal diffusion model, and monomodal for the gelator.

**Figure 5:**
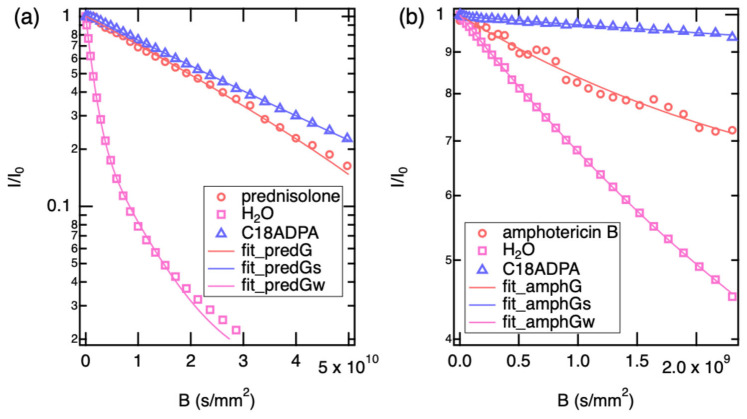
The Stejskal–Tanner plots of (**a**) prednisolone, (**b**) amphotericin B diffusion in gel samples. The diffusion data of water were fitted with bimodal diffusion model, and monomodal for the drugs and the gelator.

In the next step, we used diffusion NMR to study the localization and mobility of the pharmaceutical guests within the C18ADPA hydrogel matrix. Unlike simple aqueous solutions, the gel environment introduces restricted diffusion, obstruction effects, and specific host–guest interactions. Figure 4 and Figure 5 show the diffusion profiles of the water-soluble drugs and the sparingly soluble drugs, respectively. For water-soluble drugs, the diffusion curves are well described by a bimodal model. However, for sparingly soluble drugs, only one diffusion mode was detected. The diffusion of the supramolecular gelator (C18ADPA) was also captured in the measurements, and a single diffusion mode was found for the gelator in all samples. The resulting diffusion coefficients are summarized in Table 2.

The supramolecular gel represents a biphasic system characterized by extreme dynamic heterogeneity. The diffusion of the gelator scaffold (C18ADPA) establishes the baseline for the rigid/semi-rigid lattice. The observed diffusion coefficient (2.1–3.2 × 10^−11^ m^2^/s) is orders of magnitude lower than that of free species, reflecting the slow reptation or undulation of the self-assembled fibrillar network. The solvent water in the gel matrix exhibits biexponential diffusion behavior. The observation of two distinct diffusion modes indicates that the exchange rate between bulk-like water and fiber-associated water is slow on the NMR timescale. This is consistent with a mean exchange time, τex, that is long relative to the diffusion observation time Δ = 20 ms. In the slow-exchange limit (τ_ex_ ≫ Δ), each population diffuses independently over the observation window and two distinct components are resolved. A rough lower bound on τex can be estimated from the requirement that molecules diffusing at D_2_ ≈ 1.1–1.6 × 10^−10^ m^2^/s must not traverse a distance larger than the interfacial layer thickness (estimated ∼ 1–2 nm) within Δ: τ_ex_ > l^2^/(2D_2_) ≈ 0.3–18 ms. Since this range overlaps with Δ = 20 ms, exchange cannot be strictly ruled out on quantitative grounds alone, and intermediate-exchange contributions may lead to a mild underestimation of the true D values for the bound fraction. This caveat is acknowledged in the interpretation of the slow-mode diffusion coefficients throughout the Discussion.

The fast water fraction (Mode 1) represents solvent molecules diffusing within the interstitial pores of the gel network. Notably, this value is reduced by approximately 60% compared to pure bulk water (2.3 × 10^−9^ m^2^/s). This reduction is not primarily due to viscosity changes, but is accurately described by obstruction theory and the tortuosity factor (λ). According to the Mackie–Meares models ($\frac{D}{D_{0}}=\frac{1}{\lambda^{2}}$), the presence of the impenetrable gel fibers forces solvent molecules to traverse a curvilinear path, reducing the mean squared displacement. The observed $D/D_{0}$ ratio implies a highly entangled network with a high fractal dimension and tortuosity (λ = 1.58). This Mackie–Meares prediction (D/D_0_ ≈ 0.40) can be tested against the observed gel-phase diffusion data for each drug, treating only pore-diffusing fractions (Mode 1) and using the free-solution value as D_0_. For the three water-soluble drugs, the observed D/D_0_ ratios for Mode 1 are: 5-fluorouracil, 4.21 × 10^−10^/7.71 × 10^−10^ = 0.55; acetylcholine, 3.00 × 10^−10^/7.03 × 10^−10^ = 0.43; and paracetamol, 4.11 × 10^−10^/5.81 × 10^−10^ = 0.71. The acetylcholine value (0.43) is in close agreement with the Mackie–Meares prediction, suggesting that its fast-diffusing fraction is primarily governed by geometric obstruction. The 5-fluorouracil value (0.55) is somewhat higher than predicted, consistent with its smaller hydrodynamic size allowing more efficient pore navigation than acetylcholine. Paracetamol’s ratio (0.71) substantially exceeds the geometric prediction, suggesting that hydrogen-bonding interactions with the interface partially compensate the obstruction effect, or that exchange between populations introduces a systematic bias. For prednisolone and amphotericin B, which show only a single (slow) mode, D/D_0_ = 3.72 × 10^−11^/4.30 × 10^−10^ = 0.09 and 1.53 × 10^−10^/2.95 × 10^−10^ = 0.52, respectively. These deviations from the pure-obstruction prediction confirm that thermodynamic partitioning and interfacial interactions, not geometric obstruction alone, govern transport for these compounds.

The slow water fraction (Mode 2) corresponds to the bound hydration shell. In the hydration shell, water molecules adsorb onto the amphiphilic surfaces of the fibers and experience restricted rotational entropy and enhanced hydrogen-bonding density. This layer acts as a “viscous skin” coating the nanofibers. This structured water phase plays a dominant role in the transport of intermediate-polarity drugs (see Section 4).

For the translational dynamics of the pharmaceutical compounds, the diffusion NMR data reveal distinct transport regimes. Prednisolone exhibits a mono-exponential decay with a diffusion coefficient (3.72 × 10^−11^ m^2^/s) that is closely similar to the gelator scaffold (3.09 × 10^−11^ m^2^/s). From a thermodynamic perspective, this is consistent with a partition coefficient strongly biased toward the fiber phase. The hydrophobic steroid core likely drives the molecule to partition preferentially out of the aqueous phase and into the lipophilic interior of the C18ADPA fibers, though contributions from surface adsorption or microviscosity effects cannot be fully excluded on the basis of diffusion data alone. The drug appears to lose its independent hydrodynamic identity and moves cooperatively with the viscoelastic fluctuations of the gel matrix.

Acetylcholine and 5-fluorouracil display biexponential diffusion, signaling that these molecules exist in a dynamic equilibrium between a mobile fraction in the pores and a bound fraction. Uniquely, the slow mode of acetylcholine (2.08 × 10^−11^ m^2^/s) approaches, but does not precisely match, the gelator diffusion coefficient (∼3.1 × 10^−11^ m^2^/s), being approximately two-thirds of the gelator value. This near-coincidence is consistent with a strong but not necessarily exclusive association with the gel scaffold—most plausibly an electrostatic interaction between the quaternary ammonium cation of acetylcholine and anionic carboxylate groups on the C18ADPA headgroups. Under this interpretation, the drug transiently adsorbs as a counter-ion to the fiber surface, with its slow mode reflecting restricted surface diffusion rather than complete co-diffusion with the gel. The residual mobility relative to the gelator could reflect partial surface exchange or lateral hopping between binding sites. The fast mode (3.0 × 10^−10^ m^2^/s) represents unbound molecules diffusing through the tortuous pore network, unhindered by surface binding.

A subtle but critical physical distinction arises when analyzing paracetamol and amphotericin B. While bimodal, the “slow” mode for paracetamol (1.19 × 10^−10^ m^2^/s) is significantly faster than the gel fibers (10^−11^ m^2^/s) but is broadly consistent with the diffusivity of the interfacial water layer (1.60 × 10^−10^ m^2^/s). This indicates that paracetamol is not physically intercalated into the solid fiber (unlike prednisolone). Instead, it is trapped in the viscous “hydration shell” lining the fibers, interacting via hydrogen bonds with the interfacial water network. It undergoes hydrodynamic coupling with the solvent boundary layer rather than mechanical coupling with the solid mesh.

For amphotericin B, the macrocycle exhibits a single mode (1.53 × 10^−10^ m^2^/s) that aligns with the interfacial water or paracetamol slow mode. One plausible interpretation combines size exclusion with specific interfacial chemistry. The large hydrodynamic radius of amphotericin B may prevent it from accessing the smaller, high-tortuosity pores available to 5-fluorouracil. Simultaneously, at the gel pH of 5.37, amphotericin B is zwitterionic (pK_a1_~5.5 for its carboxyl group, pK_a2_~10 for its amine), meaning it carries both a partial anionic and a cationic center simultaneously. This zwitterionic character could promote electrostatic docking at the C18ADPA fiber surface, where the oppositely charged headgroups of the gelator provide complementary anchoring points, further confining the molecule to the structured interfacial layer. However, alternative explanations—including confinement within heterogeneous domains of intermediate viscosity, or exchange kinetics beyond the NMR timescale—cannot be ruled out on the basis of diffusion data alone.

## 4. Discussion

### 4.1. Liquid-Crystalline Dynamics of the C18ADPA Fibrillar Network

Diffusion-NMR analysis provides a unique probe into the molecular-scale dynamics of the C18ADPA hydrogel. While macroscopic rheometry characterizes the material as a viscoelastic solid (G’ >> G”), the measured self-diffusion coefficient for the gelator molecule (D_C18ADPA_ ≈ 3.1 × 10^−11^ m^2^/s) reveals surprisingly high molecular mobility [15,16,17,21]. This value provides direct quantitative confirmation of the liquid-crystalline nature of the bilayer assembly established by previous structural studies from our laboratory.

Previous studies from our laboratory have established the structural characteristics of C18ADPA assemblies at room temperature [17]. At 25 °C, C18ADPA self-assembles into liquid-crystalline lamellar bilayer structures that form branched tubular networks. X-ray diffraction reveals long-range periodic order with d-spacings of 45 Å and 35 Å (fully-interdigitated alkyl chains), characteristic of ordered lamellar phases. Upon heating above 43 °C, the system undergoes a gel-to-gel transition, transforming from these lamellar bilayers into entangled wormlike micelles. This temperature-dependent polymorphism demonstrates the dynamic, stimuli-responsive nature of the C18ADPA assembly [22].

The observed diffusion coefficient of 3.1 × 10^−11^ m^2^/s is entirely consistent with the range characteristic of fluid liquid-crystalline lamellar phases (Lα phases) of phospholipid and surfactant bilayers, providing further support for the structural assignment above. Extensive literature on lipid bilayer systems demonstrates that lateral diffusion in the Lα phase typically occurs at 10^−12^ to 10^−11^ m^2^/s (or 10^−8^ to 10^−7^ cm^2^/s) [23,24]. For example, studies on DPPC, DMPC, and DOPC bilayers in their fluid states report diffusion coefficients of approximately 2–8 × 10^−12^ m^2^/s [25,26,27]. The C18ADPA diffusivity of 3 × 10^−11^ m^2^/s is entirely consistent with these established values, confirming the liquid-crystalline state of the bilayers [28].

### 4.2. Interpreting Drug Diffusion Through Established Transport Frameworks

Before discussing individual drugs, it is useful to establish clear conceptual criteria for interpreting the two diffusion behaviors observed experimentally. Monomodal (single mode) diffusion in the gel indicates that the drug does not maintain a distinct population in the bulk aqueous pore phase. The drug exists predominantly in a single microenvironmental compartment throughout the NMR observation time. Depending on which compartment this is, monomodal diffusion can reflect either (i) deep partitioning into the bilayer interior, in which case the drug co-diffuses with the gel scaffold, or (ii) confinement at the fiber–water interface, in which case the drug diffuses at a rate characteristic of the interfacial hydration layer. Bimodal (dual-mode) diffusion, by contrast, indicates that the drug is distributed across two kinetically distinct populations, a fast-diffusing pore fraction and a slow-diffusing bound fraction, that are in slow exchange relative to the NMR diffusion observation time (Δ = 20 ms). The slow mode can then reflect either direct surface adsorption or interfacial hydrodynamic coupling, distinguishing electrostatic anchoring from hydrogen-bond-driven viscous retardation. These two criteria (the number of modes, and the identity of the reference component matched by the slow mode) provide the interpretive framework applied throughout this section.

In the Lα liquid-crystalline phase, the hydrocarbon tails of amphiphiles exhibit significant configurational entropy through rapid trans-gauche isomerizations [28]. This liquid-crystalline architecture explains several key observations in the drug diffusion data. The transport of solutes through hydrogel networks has been extensively studied and is typically analyzed using three main theoretical frameworks: the Ogston model (obstruction theory) [29], the Mackie–Meares model [30], and the free-volume theory [31]. While these models were originally developed for polymer hydrogels, they provide a useful starting point for understanding drug transport in supramolecular systems. They treat the matrix as chemically inert obstacles that retard diffusion through purely physical effects. In contrast, the liquid-crystalline C18ADPA scaffold presents chemically distinct microdomains that interact specifically with drug molecules based on polarity, charge, and amphiphilicity. By correlating observed drug diffusion coefficients with those of the gelator and water populations, we identify the most probable dominant transport mechanism for each drug. We note that all mechanistic interpretations below rely on the assumption that the liquid-crystalline bilayer architecture is preserved upon drug loading, as established for the neat gel by previous structural studies from our laboratory. Direct structural characterization of the drug-loaded systems (e.g., SAXS/WAXS) was not performed in the present work and would be required to confirm that drug incorporation does not perturb the lamellar organization or bilayer packing.

(I) Monomodal Diffusion: Bilayer Partitioning

Prednisolone exhibits monomodal diffusion (D = 3.72 × 10^−11^ m^2^/s) that closely approaches the gelator diffusion (3.09 × 10^−11^ m^2^/s) almost exactly. This coupling is most consistent with strong preferential partitioning of the lipophilic steroid into the fluid hydrophobic bilayer core, though deep surface adsorption or confinement within high-viscosity interfacial regions are alternative interpretations that cannot be excluded from diffusion data alone. If bilayer intercalation is the dominant mechanism, the drug would be dissolved within the Lα-phase interior, experiencing the same lateral diffusion environment as the surrounding C18ADPA alkyl chains. Prednisolone may thus become integrated into the liquid-crystalline assembly, participating in the two-dimensional lateral dynamics characteristic of the Lα phase.

Classical obstruction theory cannot explain this behavior: a molecule with r_H_ = 0.41 nm should diffuse orders of magnitude faster through the aqueous pore network than observed. Instead, the near-complete suppression of independent aqueous-phase diffusion strongly suggests that thermodynamic partitioning into or onto the hydrophobic bilayer fundamentally changes the diffusion mechanism from pore navigation to lateral bilayer or interfacial dynamics. The close similarity in diffusion coefficient between prednisolone and the gelator is consistent with drug mobility in liquid-crystalline hydrogels being governed by thermodynamic partitioning and chemical affinity rather than molecular size, although the precise location of the drug within the bilayer architecture would require complementary structural characterization to confirm unambiguously.

Amphotericin B also exhibits monomodal diffusion (D = 1.53 × 10^−10^ m^2^/s), but approximately five times faster than the gelator. This amphiphilic macrocycle is essentially water-insoluble yet diffuses too rapidly for complete burial in the bilayer core. The most plausible explanation is interfacial localization driven by two complementary forces. First, the amphiphilic architecture of amphotericin B (polyene backbone and polyol headgroups) favors positioning at the fiber–water interface rather than either extreme (bulk water or bilayer core). Second, at the gel pH of 5.37, amphotericin B is near its carboxyl pK_a_ (~5.5) and thus exists as a zwitterion, with its amine fully protonated and its carboxyl ~50% deprotonated. This charge complementarity with the C18ADPA surface (cationic amine interacting with anionic C18ADPA carboxylate, and vice versa) provides an additional electrostatic driving force for interfacial docking that is absent for the other four drugs. Together, these effects confine the molecule to the structured water layer adjacent to the fiber surface, enabling lateral diffusion faster than the bulk bilayer yet slower than pore diffusion. Notably, amphotericin B diffusion (1.53 × 10^−10^ m^2^/s) is intermediate between the gelator and bulk pore water, consistent with the structured water layer diffusivities observed across samples (1.1–1.6 × 10^−10^ m^2^/s), suggesting its motion couples to interfacial dynamics rather than deep bilayer partitioning.

(II) Bimodal Diffusion: Dynamic Partitioning and Interfacial Interactions

The three water-soluble drugs (5-fluorouracil, acetylcholine, paracetamol) display biexponential diffusion, indicating dynamic equilibrium between fast-diffusing populations in bulk pore water and slow-diffusing populations experiencing retardation through different mechanisms.

Acetylcholine’s slow mode (D_slow_ = 2.08 × 10^−11^ m^2^/s) approaches the gelator diffusion coefficient, suggesting strong surface association—most likely through Coulombic attraction of the quaternary ammonium group to anionic C18ADPA carboxylates, creating a transient adsorption state. As noted above, the slow mode is approximately two-thirds of the gelator value rather than an exact match, which may reflect partial surface exchange or lateral hopping along the fiber [32]. The fast mode (D_fast_ = 3.0 × 10^−10^ m^2^/s) represents unbound molecules diffusing through tortuous pores. This two-site exchange behavior (D_obs_ = f_bound_ × D_gel_ + (1 − f_bound_) × D_pore_) is consistent with reversible, relatively weak surface association (binding energies of this type are typically a few k_B_ T), allowing rapid association–dissociation kinetics on the NMR timescale, consistent with the bimodal diffusion observed [33].

In contrast, paracetamol and 5-fluorouracil exhibit slow modes (1.19 × 10^−10^ and 8.43 × 10^−11^ m^2^/s) that fall well above the gelator diffusivity but below the bulk pore water values, placing them within the diffusivity range characteristic of the structured interfacial water layer. These neutral polar molecules do not bind directly to the fiber surface but partition into the viscous interfacial water layer, where their motion couples hydrodynamically to the local solvent dynamics. The gel fibers are surrounded by a hydration shell with 2–5 times higher viscosity than bulk water due to hydrogen bonding with gelator headgroups. Drugs in this layer experience viscous drag that retards diffusion without direct binding to the solid scaffold.

The variation in slow-mode diffusion among the three drugs reflects their specific interactions with the interfacial region. Acetylcholine’s electrostatic binding produces the most pronounced retardation (approaching gelator dynamics). Paracetamol’s hydrogen-bonding capability results in intermediate retardation (matching bound water). A consideration of each drug’s ionization state at the gel pH of 5.37 is essential for mechanistic interpretation. Acetylcholine carries a permanent positive charge at all pH values (quaternary ammonium cation), making electrostatic interaction with the anionic C18ADPA carboxylates a certainty. Paracetamol (pK_a_~9.5) and 5-fluorouracil (pK_a1_~7.5) are both effectively fully neutral at pH 5.37 (>99% neutral form by Henderson–Hasselbalch), ruling out charge-based explanations for their behavior. The faster slow-mode diffusion of 5-FU relative to paracetamol is better attributed to its smaller size and fewer persistent hydrogen-bond groups. Prednisolone has no ionizable groups whatsoever and is a neutral non-electrolyte at all pH values, consistent with pure hydrophobic partitioning. Notably, amphotericin B is the only drug among the five with ionizable groups near the gel pH (pK_a1_~5.5 for the carboxyl, pK_a2_~10 for the amine), meaning it exists as a zwitterion at pH 5.37: the amine is essentially fully protonated (+1) while the carboxyl is ~50% deprotonated. This zwitterionic character provides an additional mechanistic explanation for its interfacial localization: electrostatic complementarity between the partial anionic carboxylate of amphotericin B and the cationic protonated amine of C18ADPA, and vice versa, could cooperatively anchor it at the fiber–water interface. This pH-driven zwitterionic interfacial trapping is a mechanistic feature unique to amphotericin B among the drugs studied.

### 4.3. Anomalous Solvent Structuring in Prednisolone-Loaded Gels

A striking feature of the prednisolone-loaded system is the anomalous retardation of the bound water fraction (Water Mode 2). Prednisolone is a neutral non-electrolyte with no ionizable groups, meaning its interaction with the gel network is driven entirely by hydrophobic and van der Waals forces—consistent with deep bilayer partitioning rather than surface electrostatic adsorption. While other formulations exhibited bound water diffusion coefficients in the range of 1.1–1.6 × 10^−10^ m^2^/s, the prednisolone sample displayed a significantly reduced value of 8.38 × 10^−11^ m^2^/s. This 50% reduction in solvent mobility provides secondary evidence for the deep intercalation of the steroid into the gel fibers. Drawing an analogy to the behavior of cholesterol in phospholipid membranes, the rigid, planar steroid backbone of prednisolone likely induces a “condensing effect” on the C18ADPA alkyl chains, reducing their configurational entropy and increasing the local ordering of the fiber surface.

The dynamics of a hydration shell are coupled to the fluctuations of the substrate. A more rigid, ordered hydrophobic surface induces a stronger hydrophobic hydration effect, where water molecules form a longer-lived, highly structured clathrate-like network. Consequently, the water molecules at the interface of the prednisolone-stiffened fibers are held more tightly than those surrounding the more fluid fibers of the other formulations. This results in a bound water population that is kinetically distinguishable, diffusing at a rate closer to that of the solid lattice itself.

## 5. Conclusions

In this study, diffusion NMR was employed to resolve the molecular-scale host–guest dynamics within a C18ADPA supramolecular hydrogel. The gelator self-diffusion coefficient (~3.1 × 10^−11^ m^2^/s) confirms that the fibers behave as a viscoelastic liquid-crystalline mesophase rather than a rigid network. A key finding is that the gel pH (5.37) is not merely a preparation parameter but an active determinant of transport: acetylcholine is permanently cationic and electrostatically immobilized at the fiber surface; 5-fluorouracil and paracetamol are neutral and retarded by hydrogen-bonding capacity alone; prednisolone is a neutral non-electrolyte that partitions into the hydrophobic bilayer core; and amphotericin B is uniquely zwitterionic at this pH, providing additional electrostatic anchoring at the fiber–water interface beyond its amphiphilic character. Collectively, these findings demonstrate that drug mobility in a supramolecular gel is governed not by molecular size alone, but by the interplay of hydrophobicity, hydrogen-bonding capacity, and pH-dependent charge state. Since these interpretations rest solely on diffusion coefficients, they remain mechanistically suggestive rather than conclusive. Definitively resolving the molecular-scale dynamics in such a system is inherently challenging, as most complementary techniques, including structural methods (SAXS etc.) and ensemble binding assays, report on static or time-averaged properties and cannot directly access the exchange kinetics and diffusion mechanisms probed here. The diffusion NMR framework presented is therefore best regarded as a hypothesis-generating foundation for future targeted investigations.

## Figures and Tables

**Table 2:** Diffusion coefficients of the drugs, the gelator (C18ADPA) and water in gel samples. Mode 1 is the fast diffusion and Mode 2 for slow diffusion.

	5-Fluorouracil	Acetylcholine	Paracetamol	Prednisolone	Amphotericin B
**Mode 1 for drug**	4.214 × 10^−10^	3.004 × 10^−10^	4.110 × 10^−10^	-	-
**Mode 2 for drug**	8.434 × 10^−11^	2.084 × 10^−11^	1.186 × 10^−10^	3.719 × 10^−11^	1.528 × 10^−10^
**C18ADPA**	3.242 × 10^−11^	3.165 × 10^−11^	2.127 × 10^−11^	3.094 × 10^−11^	2.314 × 10^−11^
**Mode 1 for water**	7.551 × 10^−10^	7.833 × 10^−10^	9.833 × 10^−10^	6.505 × 10^−10^	6.393 × 10^−10^
**Mode 2 for water**	1.129 × 10^−10^	1.465 × 10^−10^	1.608 × 10^−10^	8.378 × 10^−11^	1.096 × 10^−10^

## Data Availability

The data that support the findings of this study are available from the corresponding author upon reasonable request.

## Supplementary Materials

The following supporting information can be downloaded at: https://www.mdpi.com/article/10.3390/pharmaceutics18050592/s1, Figures S1–S5: PGSE NMR diffusion analysis of the drugs in C18ADPA hydrogel.
