# Supplementary material for: Drug Transport in a Liquid-Crystalline Supramolecular Hydrogel: Diffusion Mechanisms Revealed by PGSE NMR
Source: Pharmaceutics. 2026 May 12;18(5):592. doi: 10.3390/pharmaceutics18050592 (PMC13210933; doi:10.3390/pharmaceutics18050592)
Supplement: Supplementary file 1 [file pharmaceutics-18-00592-s001.zip › pharmaceutics-4268965-supplementary.pdf]

Supporting information for

Drug Transport in a Liquid-Crystalline Supramolecular Hydrogel: Diffusion Mechanisms Revealed by PGSE NMR

Wei Wang [1,2]

[1] Department of Chemistry, University of Bergen, 5020 Bergen, Norway; wei.wang@uib.no; Tel.: +47-55583355

[2] Centre for Pharmacy, University of Bergen, 5020 Bergen, Norway

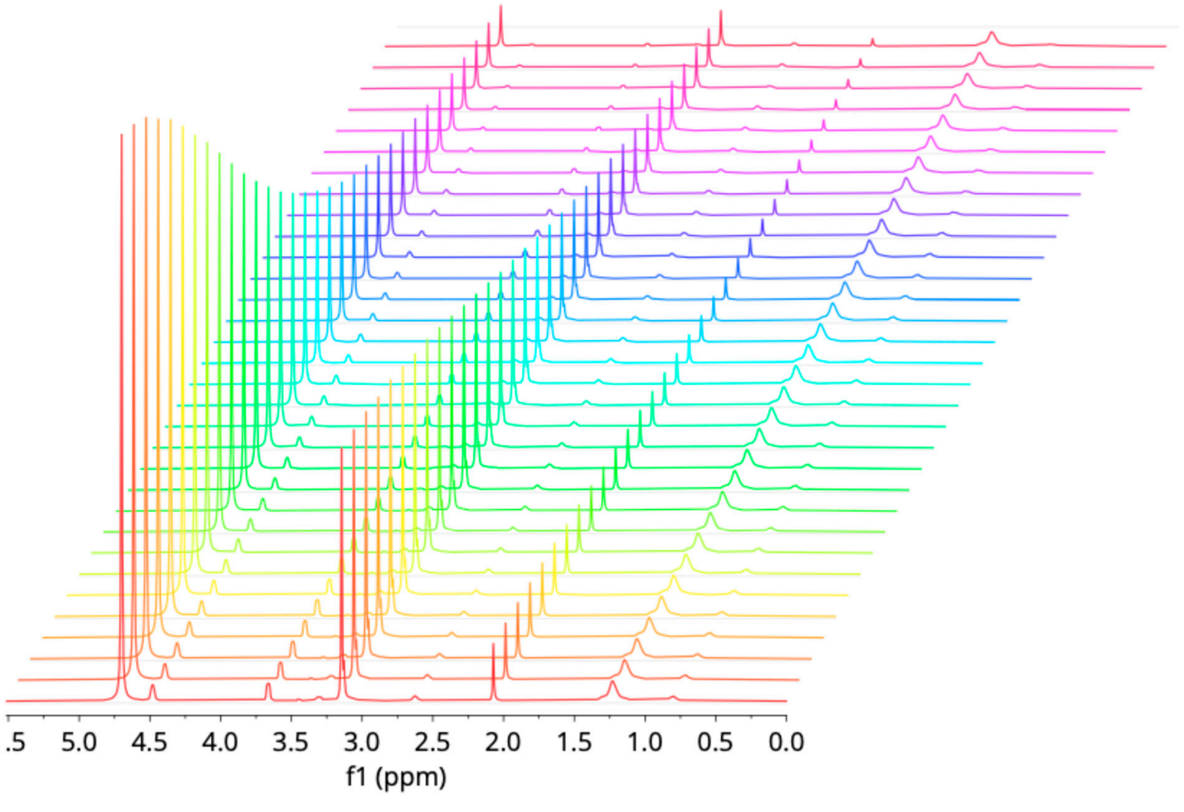

Figure S1. PGSE NMR diffusion analysis of acetylcholine in C18ADPA hydrogel.

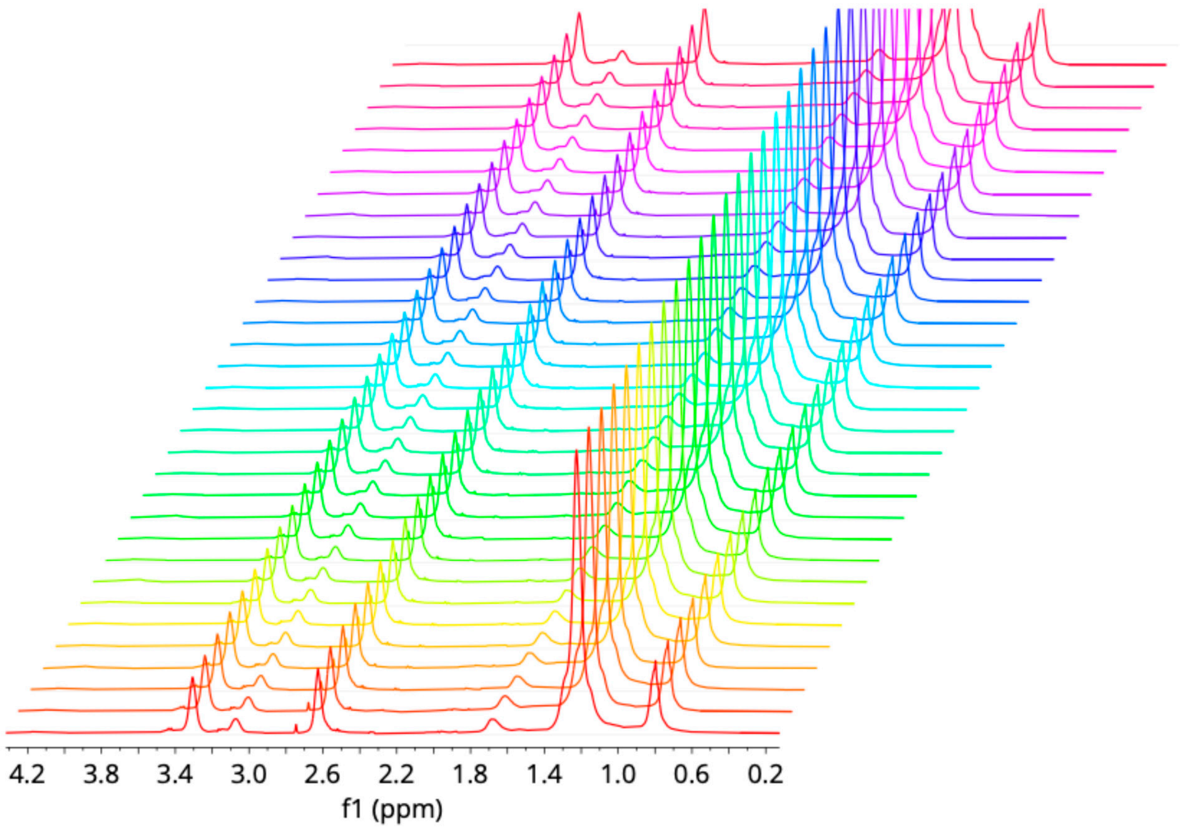

Figure S2. PGSE NMR diffusion analysis of amphotericin B in C18ADPA hydrogel.

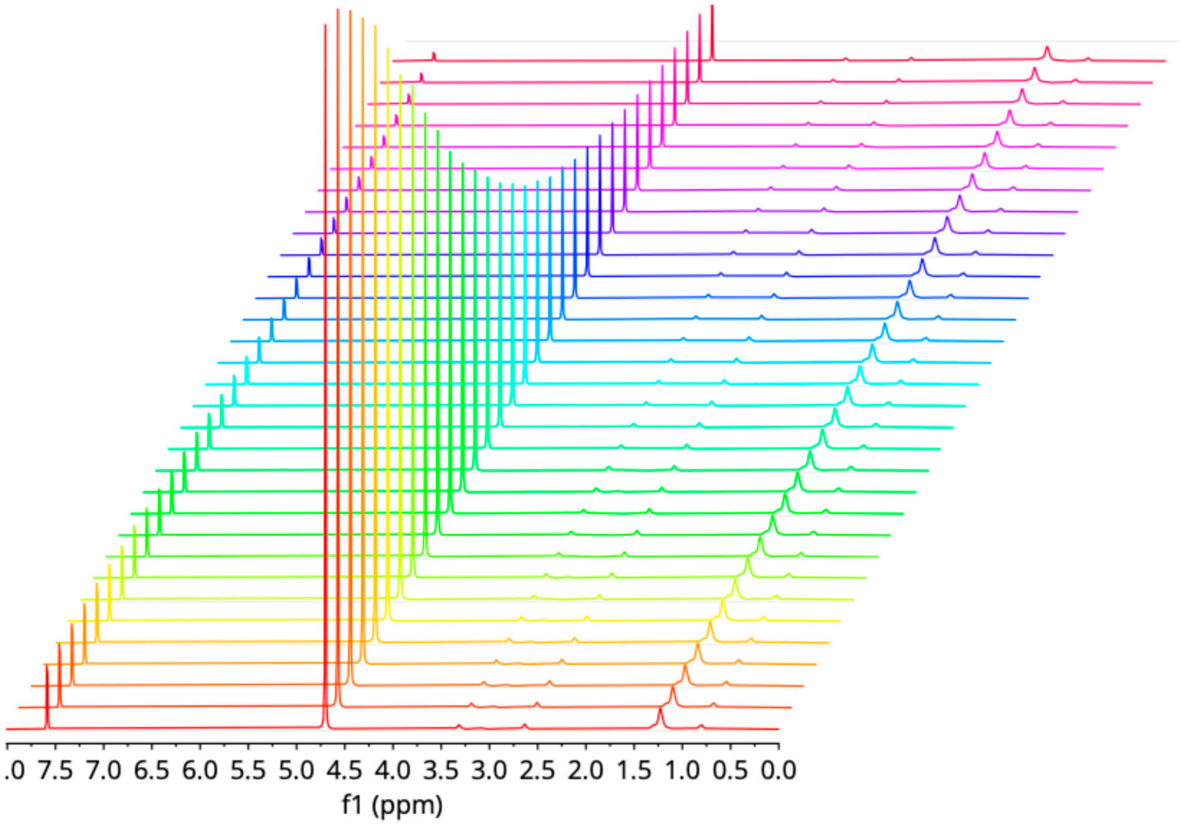

Figure S3. PGSE NMR diffusion analysis of 5-fluorouracil in C18ADPA hydrogel.

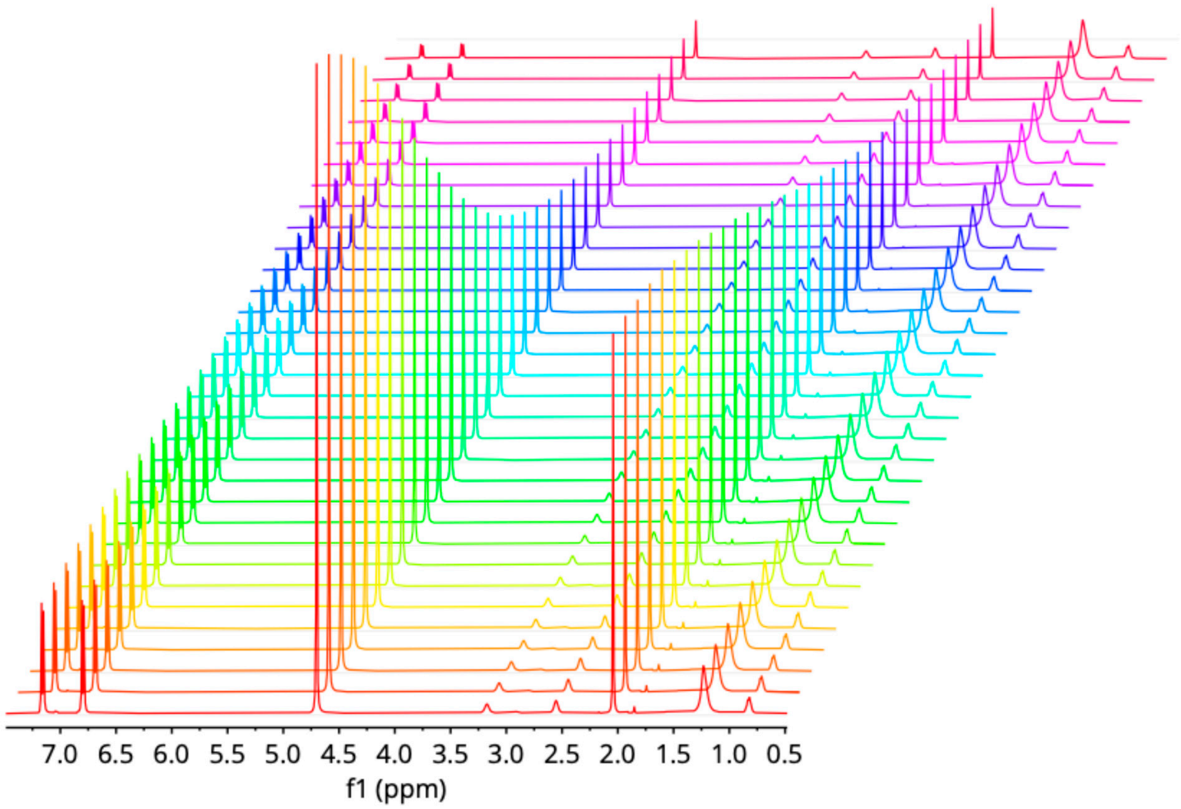

Figure S4. PGSE NMR diffusion analysis of paracetamol in C18ADPA hydrogel.

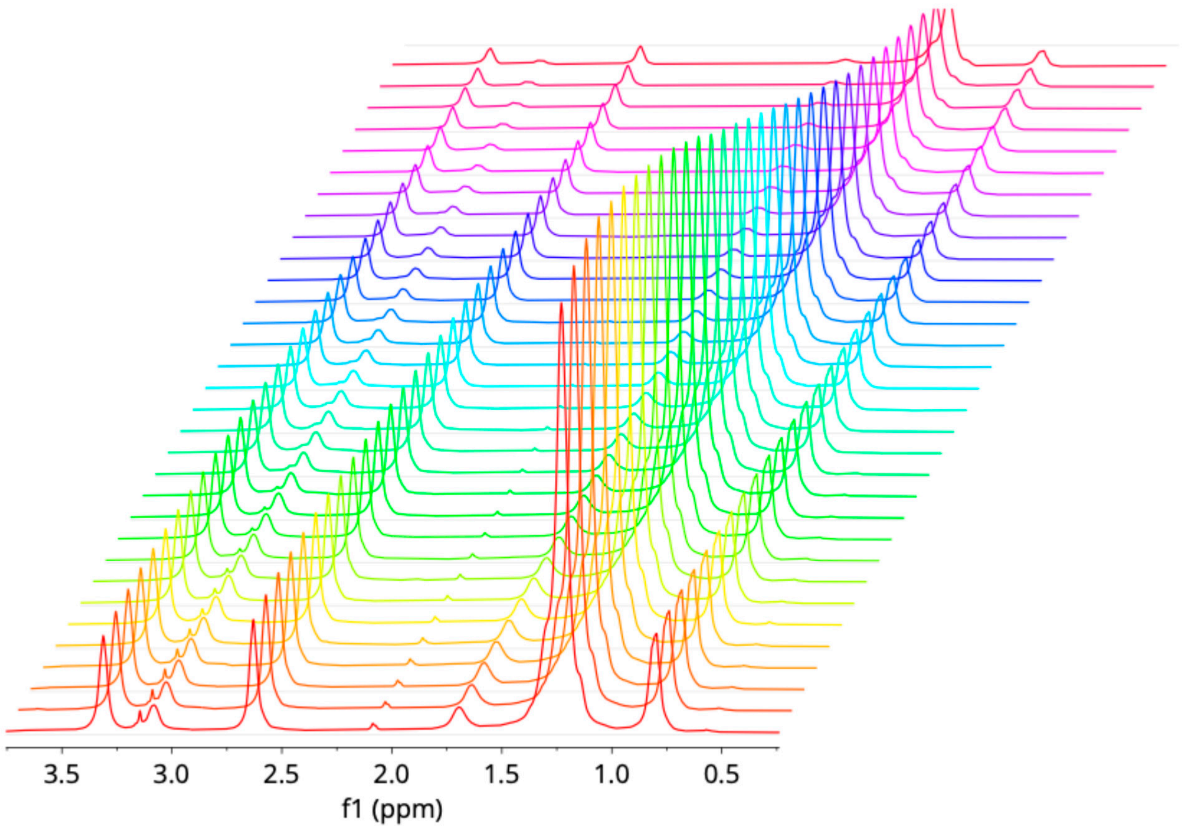

Figure S5. PGSE NMR diffusion analysis of prednisolone in C18ADPA hydrogel.
